# In Memoriam

**Published:** 1863-06

**Authors:** 


### IN MEMORIAM.

MORE than thirty years ago, we had a neighbor in the far, far West, on the banks of the turbid Missouri, when St. Louis itself did not number seven thousand inhabitants. He was a man of culture, refinement, and an elevation of character which commanded the respect and deference of all who knew him. Even then "gray hairs were upon him," and in his face were those lines of firmness of purpose, of past care and responsibility, which carried with them an air of authority; and yet every thing about him indicated the polished gentleman; and in the course of time these characteristics, modified by the benign influences of the Christian religion, grew into so much that was kind and loving and benignant, that we can not resist the inclination to tell the story of his life to our readers, in the hope that those of them who are young may pattern after his firmness of purpose, his integrity, his temperate life, and, like him, live to a thankful old age; for, writing on his seventieth birthday, he says:

"I am certainly becoming more and more infirm of body, from the effects of advancing age. However this may be, sure I am that God hath dealt with me through the past year, as well as through *all* my past life, in great mercy and kindness; spiritually, I do hope more than ever before; temporally, without any diminution of former allowances of comfort and abundance. Altogether, I may truly say, and I *do* say, 'my cup runneth over.' O Lord! give me grace to respond with becoming gratitude to thee for all thy favors so largely conferred on thy unworthy servant, my family and household, and constrain me to live out my earthly pilgrimage consistently with my obligations of love and duty to thee, and consistently with what I well know to be for my true happiness, here and forever. I humbly ask this, my Father, for my Redeemer's sake."

As he grew older, the kindliness of his nature grew upon him, and his whole heart was set on devising some method by which he could, in the largest way, according to his means, benefit those who might come after him. The plan which seemed most feasible for him, was founding an institution of learning, the nature of which is indicated in an extract from the deed conveying one hundred and twenty acres of land adjoining a large and thriving town, for the endowment of Lindenwood Female College, executed July the fourth, 1856:

"The Lindenwood Female College is to be set up and established on a large and liberal plan, and on a lasting foundation, to consist of primary, high, and normal schools, with a domestic and boarding department connected therewith. It is to supply, at as low charges as practicable, ample facilities for female education in the best sense [meaning] of the term, the proper development and cultivation of the intellectual, moral, and physical faculties. It is to present a school or schools wherein female youth, given in baptism to the Redeemer, (not excluding others,) may be properly educated and qualified for the important duties of Christian mothers and school-teachers; wherein the Holy Bible shall always have a prominent place, and be a permanent class-book; in which the whole course of instruction and discipline shall be based on the religion of Jesus Christ, as held and taught in the Confession of Faith and Catechisms of the Presbyterian Church in the United States of America, adopted by the General Assembly of said Church in the year 1821. In fine, to supply schools adapted to qualify the pupils not only to enjoy the rational pleasures of life as accountable beings, but to become enlightened, accomplished, and useful members of society; to discharge with ease and grace the peculiar duties of the sex in all their various relations; also, so to convey and adapt instruction appropriately, as to give a decidedly *national* bias to the youthful mind."

The place known as "Lindenwood," near St. Charles, Missouri, was purchased by the donor upward of fifty years ago, being all that was left of a large tract of landed estate owned by him previous to becoming security for two young men who had been his clerks in the Indian Department, by which he became indebted to the Government to the amount of twenty thousand dollars, to pay which he surrendered all his property; Lindenwood was the surplus. He was upward of twenty years in the Indian Department, and as Commissioner to open and survey the road from the then western boundary of the United States to Santa Fé, in New-Mexico. He then retired to Lindenwood and erected a log-cabin in 1828, under the shade of the noble Linden trees which flourished there; and there he lived when we first met him and his noble wife in 1832; and there he continued to live until 1859, when the homestead was given up for the use of the "Lindenwood College," which was commenced in 1830 as a small school in a log-cabin, by the wife, whose pupils were chiefly composed of her sisters, nieces, and a few children of her intimate friends. There were soon more applications than the log-cabin would accommodate. An addition was then built: teachers from the East were employed, and the Institution continued, with varying success, until it passed into other hands in the shape of a regularly organized college.

It will, no doubt, have been anticipated by the reader that such a character as has been described must have descended from the sterling old Puritan stock of the May-Flower days; and so it was, for George Champlin Sibley, of Lindenwood,* Missouri, farmer, was born in Great Barrington, Berkshire county, Massachusetts, on Monday morning, the first day of April, 1782, and died January 31st, 1863, aged eighty-one years. His father was the late Dr. John Sibley, of Natchitoches, Louisiana, and the third of the fifteen children of Colonel Timothy Sibley, of Sutton, Massachusetts, who was the fifth son of John Sibley, of Salem, who died in 1754, aged ninety-five, who was the eldest son of John Sibley, a native of England, who,

* It may as well be stated here that this beautiful place received, most appropriately, its present name long before Mr. Van Buren's Lindenwald was known. It was so called simply for the reason that numerous clumps of the Linden, strikingly large and beautiful, are the natural forest growth of the spot now occupied by the homestead, and are carefully preserved.

with his brother Ebenezer, came to America in 1640, and settled in or near Salem. The brothers were of the Friends, and adherents of Oliver Cromwell, John Hampden, John Pym, and held religious and political opinions and views very decidedly of the Puritanic and anti-despotic parties of the day. It was to escape from the intolerant government and cruel policy of Charles the Fifth and Archbishop Laud, that they left their native land to seek an asylum from civil and religious oppression in the new settlements of "North-Virginia," now better known as "New-England." They and their associates were actuated and impelled by the same true and lofty principles of liberty and right that freighted the May-Flower in 1620. John, of England, died in 1710, aged ninety-six; Colonel Timothy Sibley, it should be noted, died in Sutton in 1819, aged ninety-two.

The mother of George C. was Elizabeth, the eldest daughter of the late Rev. Samuel Hopkins, D.D., of Newport, Rhode Island, who, as it is recorded, "was the direct lineal descendant of Stephen Hopkins, one of the blessed men who landed at Plymouth in December, 1620." Thus it appears that the subject of this memoir inherited Puritan blood through both lines of his parentage; and this he has ever claimed, together with the legitimate concomitant principles in their fullest sense.

Dr. John Sibley entered the Revolutionary army in the medical staff in 1777, being then scarcely "of age," in which capacity he served throughout the struggle with unflinching fidelity and zeal. He married Elizabeth Hopkins at Great Barrington in 1780. Two sons were the issue, George Champlin and Samuel Hopkins; the latter was born at Newport, 16th April, 1784; died at Natchitoches, Louisiana, 17th November, 1823, leaving a widow and four children, two sons and two daughters. Of those children two only are now living—Elizabeth, the wife of Colonel Francis Lee, U.S.A., and Major Henry Hopkins Sibley, also of the army; these both served under General Scott in the late war with Mexico, and were both breveted (Colonel L. twice) for brilliant and meritorious services.

As soon after the close of the Revolutionary contest as he could arrange his private affairs, Dr. Sibley settled in Fayetteville, North-Carolina, with his family, in the practice of his profession. On the 25th October, 1790, his wife Elizabeth died. This sad bereavement fell heavily on the Doctor and his two little sons, one nine, the other seven years old. Mrs. Elizabeth Hopkins Sibley was, in the truest sense, an accomplished lady — an accomplished *Christian* lady — and was such a wife and mother as very few have ever excelled. . . . Dr. Sibley continued to reside in Fayetteville till soon after the acquisition of Louisiana, when he removed to Natchitoches, on the Red River, at which place he died 8th April, 1837, in the eightieth year of his age.

From a long obituary that appeared in the *Natchitoches Herald* of the 13th April, 1837, the quotation of a few paragraphs may be here allowed:

"At different periods of his life, Dr. Sibley was intrusted (unsolicited by him) with delicate and important duties by the General and State Governments, and also by the voters of the Senatorial district in which he resided; and never have we heard expressed the least dissatisfaction as to the discharge of those duties. His death will be long and deeply lamented here. Our town has lost its chief ornament, the aged have lost a cheerful companion, the young an excellent example of what a gentleman should be, and the poor a friend whose ear was never shut against a tale of distress, who ever sympathized with them in their sufferings, and whose purse was ever open to relieve the destitute.

"In private life Dr. Sibley was a devoted husband, a tender parent, a warm and faithful friend — of unblemished integrity, of lofty and honorable sentiments—affable in his manners, easy of access to those who sought advice and instruction. He was gifted with rare colloquial powers, and was enabled (enriched as was his mind with the experience, observation, and carefully hived learning of a long and well-spent life) not only to give zest to the pleasures of rational conversation, but to impart much curious and valuable knowledge to be derived from no other source." .

The education proper of Major Sibley commenced practically almost in his babyhood, though his "school-days" were not ended till he had nearly attained his nineteenth year. At the feet of his pious Christian mother, lessons were taught and impressed that neither time nor eternity can eradicate. Such lessons, from such lips, at such an age, are invaluable. They may sometimes, in the bustle of life, be more or less unheeded for a time, but their legitimate office is, to give the true, reliable stamina to character, and they seldom fail to do so.

When the good and the gifted die, especially in a serene old age, as Major Sibley did, it may be practically useful to look at some of the circumstances which contribute to such results—to wit, a Christian and kindly old age; an old age whose predominating moral quality was that humble, habitual thankfulness toward the GIVER of all good, indicated in the diary extracts quoted above, or as expressed in some of the verses of glorious Isaac Watts:

> "Unnumbered comforts to my soul
> Thy tender care bestowed,
> Before my infant heart conceived
> From whom those blessings flowed."

But a greater blessing than any thousand of life's comforts, is

> ———"a cheerful heart
> That tastes those gifts with joy;"

that acknowledges them; that receives them as Major Sibley did, with a sense of unworthiness, and an all-absorbing gratitude and love.

While the life within was that of sunshine and thanksgiving the physical frame was maturing, as a shock of corn, for the ripeness of immortality — still lingering on the shores of time beyond the age of fourscore years.

Steady force of character, with determination, are of more value toward insuring longevity than a good constitution. Men of "purpose" can live down disease; can live above it, as did the hero of Macaulay's *History of England*, William the Conqueror, who was a wheezing asthmatic all his life, and he died at last by being thrown from his horse.

The dogged determination to accomplish a business for which he was sent is illustrated in the latter part of the following narration, taken from a late *National Intelligencer*, the editor of which was the personal friend of Major Sibley:

"The years of his childhood and youth were spent in North-Carolina, from whence, while yet quite a youth, he was appointed by Mr. Jefferson to an office in the Indian Department, and sent to St. Louis soon after the purchase of Louisiana, and arrived there after Gen. Wilkinson had taken formal possession of the country as U. S. Military Governor. Not long after this he was sent into the Indian country as Indian Agent and factor. It was at this period that he went out with a hundred Osage warriors, and visited the Grand Saline and Salt Mountain, and explored a region of country which he supposed had never before been seen by the white man. A report of the wonders of nature he there saw has been already published. His many letters written during his Agency to the Government attest his interest in the welfare of the red men of the West, as well as his untiring devotion to the duties of his station. Those duties he performed with honor to himself and satisfaction to his Government. As late as the year 1841 he wrote a long letter to Henry Clay on Indian affairs, which, if the advice therein contained had been heeded, the late dreadful scenes in Minnesota would never have taken place. Soon after he retired from the Indian Department he was appointed one of the three Commissioners to survey and mark out a road from Missouri to New-Mexico. He was the only one of the three who went to Santa Fé, where, and at Taos, he remained a year with the surveyor and party before permission could be obtained from the Mexican Government to survey and mark the road through its territory. This duty, which involved some treaties with the Indians, was performed with his usual zeal and fidelity."

Major S. was a man of strictly temperate habits; for the last twelve years of his life he never took any medicine, nor did he drink any thing stronger than black tea or weak coffee.

Who will deny that quite as much as firmness of purpose and a temperate life, a long and consistent love for the Bible and the practice of its teachings, with a heart steadily feeding on endeavors to promote the welfare of others, have an influence in protecting life to over fourscore years; and, in fact, this is the truest philosophy in connection with that greatly coveted ability of "retiring from business," that while a man ceases to occupy his powers, his experience, his talents in monetary affairs, he should at once begin to employ them in a new direction, with scarcely less energy than when in the whirl and turmoil of the exchange, the counting-room, and the street; in the direction of planning and setting in motion organizations for happifying and elevating the great brotherhood of man; these bring out the business energies in a new connection, in a connection not where dollars and cents and self-interest harden and contract and defile all that is touched, but a connection which brings into hourly exercise the nobler part of man, his pity for the poor, his sympathy for the wronged, his benevolence toward all. Thus we find Major Sibley consecrating his later years to temperance, to the cause of African colonization, to the spread of the Bible, and to the early and proper education of girls. Says the *National Intelligencer*, "He was a great friend to African colonization;" and it was in this connection that the very day before he died the last effort of his pen was to write and forward an article on slavery to *The Presbyterian*, of Philadelphia, in as fair and beautiful and steady a hand as he would have written at the age of twenty-five. Dying with his harness on, working for humanity to the very last. He was the friend, the promoter and advocate of the Bible cause, having been for many years, and continued to be until his death, President of the St. Charles County Bible Society of Missouri. Major Sibley was also a zealous advocate of Christian education, as is proved by the standing monument, "Lindenwood College," erected (by subscription) on a tract of land which he had owned for nearly fifty years, and on which he lived over thirty years—the most beautiful, the best improved, the most valuable part of which, amounting to one hundred and twenty acres, with the hearty consent of his wife, he gave to the College.

But we are unwilling to close this article without naming another influence which made itself felt for good in forming the whole character of Major S. from early manhood, to wit, from the day he was married, in 1815, to Mary, eldest daughter of Col. Rufus Easton, at that time a delegate to Congress from the Territory of Missouri. They never had any children. For nearly fifty years she was an angel of light to him. She had herself an energy of character of such a firm, steady, Christian consistency of purpose, that in the course of years it won her husband over to embrace the same religion, to feed upon the same hopes of immortality, and to rest on the same foundation-stone upon which she herself grounded all her anticipations of a glorious existence beyond the grave. The means which she used to bring him over to the faith once delivered to the saints, were mainly two, one secret, the other open and known of all men. Secret prayer, and a life of patient, consistent, unswerving piety *extending through many years;* but glorious fruit came at last! May many of the young gentlemen who read this article be so fortunate as to obtain such a wife as Mary Sibley was; may many a mother from this hour resolve that she will educate her daughters to perform as well the duties of an educated, pious wife.

